# Adaptability in Swimming Pattern: How Propulsive Action Is Modified as a Function of Speed and Skill

**DOI:** 10.3389/fspor.2021.618990

**Published:** 2021-04-07

**Authors:** Christophe Schnitzler, Ludovic Seifert, Chris Button

**Affiliations:** ^1^Laboratory Equipe d'accueil en Sciences Sociales (E3S, UR1342), Faculté des Sciences du Sport, Université de Strasbourg, Strasbourg, France; ^2^Cetaps EA3832, Faculty of Sport Sciences, University of Rouen, Normandie, France; ^3^School of Physical Education, University of Otago, Dunedin, New Zealand

**Keywords:** motor control, expertise, force, coordination, spectral analysis, constraint-led approach

## Abstract

The objectives of this study were to identify how spatiotemporal, kinetic, and kinematic parameters could (i) characterize swimmers' adaptability to different swimming speeds and (ii) discriminate expertise level among swimmers. Twenty male participants, grouped into (a) low-, (b) medium-, and (c) high-expertise levels, swam at four different swim paces of 70, 80, 90% (for 20 s), and 100% (for 10 s) of their maximal speed in a swimming flume. We hypothesized that (i) to swim faster, swimmers increase both propulsion time and the overall force impulse during a swimming cycle; (ii) in the frequency domain, expert swimmers are able to maintain the relative contribution of the main harmonics to the overall force spectrum. We used three underwater video cameras to derive stroking parameters [stroke rate (SR), stroke length (SL), stroke index (SI)]. Force sensors placed on the hands were used to compute kinetic parameters, in conjunction with video data. Parametric statistics examined speed and expertise effects. Results showed that swimmers shared similarities across expertise levels to increase swim speed: SR, the percentage of time devoted to propulsion within a cycle, and the index of coordination (IdC) increased significantly. In contrast, the force impulse (*I*^+^) generated by the hand during propulsion remained constant. Only the high-expertise group showed modification in the spectral content of its force distribution at high SR. Examination of stroking parameters showed that only high-expertise swimmers exhibited higher values of both SL and SI and that the low- and high-expertise groups exhibited similar IdC and even higher magnitude in *I*^+^. In conclusion, all swimmers exhibit adaptable behavior to change swim pace when required. However, high-skilled swimming is characterized by broader functional adaptation in force parameters.

## Introduction

Three main categories of constraints shape human movement behavior, namely, the task (which refers to the task goal), environmental (physical variables in which the behavior takes place), and organismic constraints (which refers to the person's characteristics) (Newell, 1986). When constraints change, behavior changes accordingly. This study seeks to identify how stroking and kinetic parameters could characterize swimmers' adaptability to four different swimming speeds but also discriminate swimming expertise. As stated by Newton's second law, for a body with a constant mass, the acceleration undergone by this body is proportional to the resultant of the forces and inversely proportional to its mass. In swimming, moving forward requires the generation of propulsive forces (*F*_prop_). However, water is a dense material (800 times more than air), and moving an object in water generates in return a drag force (*F*_drag_) proportional to its speed. In the case of human bodies, the relationship between *F*_drag_ and swim speed can be approximated according to Equation (1) (Toussaint and Truijens, 2005).

(1) $$\begin{array}{r} F_{drag}\;\sim \;{\text{k}\cdot \text{S}\cdot \text{V}}^{1.8-2.2} \end{array}$$

where *k* is coefficient related to body shape; *S*, surface presented toward direction of travel in m^2^; *V*, speed in m s^−1^.

When swimming at a constant speed, Equation 2 applies, according to Newton first law:

(2) $$\begin{array}{r} F_{prop}\;=\;F_{drag} \end{array}$$

The implication is that when swimming fast, *F*_drag_ is high, and *F*_prop_ has to be scaled up accordingly.

Within the swim cycle, speed fluctuations occur (Schnitzler et al., 2010, 2011b; Barbosa et al., 2013), as *F*_prop_ is generated by arms and legs, which act at different moments within the swim cycle. In front crawl, the total propulsive time during one complete cycle is composed of two propulsive phases performed by the arms (one per arm, subdivided into pull-and-push phase), with time gap, continuity or superposition between those propulsive actions, and multiple leg beat kicks (typically two to six) (Chollet et al., 2000). Each of these propellers generates force over a short duration within the cycle, called propulsive impulses. Mathematically, an impulse represents the time integral of the resultant force acting on a body (Robertson et al., 2014). According to Alberty et al. (2009) over a swim cycle, the force impulse, *I*^+^ (N · s), is the integral over time of the total force production (Equation 3).

(3) $$\begin{array}{r} I^{+}=\int_{t2}^{t1}F\left(t\right)dt \end{array}$$

with *dt* corresponding to the propulsive time duration.

Considering propulsive impulse only, Equation 4 applies

(4) $$I^{+}=n\times ({I^{\text{+/}}}_{\text{rightarm}}+{I^{\text{+/}}}_{\text{leftarm}}+{I^{\text{+/}}}_{\text{rightleg}}+{I^{\text{+/}}}_{\text{leftleg}})$$

n: number of cycles during the period considered

$I_{\text{rightarm}}^{\text{+/}}+I_{\text{leftarm}}^{\text{+/}}+I_{\text{rightleg}}^{\text{+/}}+I_{\text{leftleg}}^{\text{+/}}$: discrete impulses from arms (right and left) and legs (right and left) during a swim cycle.

Over this period of n cycles of period T, Equation 5 defines average force

(5) $$\begin{array}{r} F_{av}=\;\sum_{i=1}^{n}I\left(i\right)\;+/\text{T} \end{array}$$

where *F*_av_ is average force; *I*(*i*)+ is force impulse over the *i*th swim cycle; *T* is duration of a swim cycle.

But only part of the force in Equation 5 generates propulsion. Studies analyzing fluid dynamics showed that part of this force provides kinetic energy to the water (Kudo et al., 2013). Hence, Toussaint et al. (1990) proposed to separate the total power output *P*_tot_ into two components: the power to overcome drag (*P*_*d*_), and the power wasted in giving a kinetic energy change to the water (*P*_*k*_), according to Equation (6).

(6) $$\begin{array}{r} {\text{P}}_{\text{tot}}\;=\;P_{d}\;+\;P_{k} \end{array}$$

As power is a linear combination of force and speed, Equation 7 also applies:

(7) $$\begin{array}{r} F_{av}=\;F_{d}\;+\;F_{k} \end{array}$$

where *F*_av_ is the average force exerted by the swimmer, *F*_d_ is force to overcome drag, and *F*_k_ is force wasted in translating kinetic energy to move the water.

Should a swimmer need to increase his/her pace, this will impact upon the required force production as mechanical power output increases with pace (Toussaint and Truijens, 2005; Seifert et al., 2011). In that, when swimming at a faster pace, *F*_d_ and *F*_av_ have to be scaled accordingly. According to Robertson et al. (2014), there are four ways of making such adaptations: (a) by increasing the amplitude of the individual force impulses, (b) by increasing the duration of individual force impulses, (c) by increasing both amplitude and duration, and (d) by increasing the frequency of the individual impulses.

Both task (i.e., to swim as fast as possible over a fixed distance) and environmental constraints (i.e., the drag directly linked to the associated swim speed) influence swim adaptation. However, task and environmental constraints are only part of the explanation when studying swimmers' behavior, as different levels of adaptability can be observed. Adaptability relates to a subtle blend between stability (i.e., persistent behavior) and flexibility (i.e., variable behavior) (Seifert et al., 2014). Adaptability is a key feature of dexterity (Bernstein, 1996), which can be defined as the expertise to reach the goal of a task correctly, quickly, rationally, efficiently, and with resourcefulness. In competitive swimming, adaptability refers to the ability to modify the coordination to swim efficiently at different paces (Simbaña-Escobar et al., 2018). Highly skilled swimmers exhibit high stroke length (SL) and stroke index (SI), with both parameters linked to swimming efficiency (Costill et al., 1985; Toussaint and Truijens, 2005). To examine swimmers' adaptability, scanning tasks in which swim speed is incremented are often proposed (for example: Schnitzler et al., 2010, 2011a; Seifert et al., 2011; de Jesus et al., 2016). The literature reveals that this adaptability may occur at different levels, as both intralimb and interlimb coordinations are affected (Guignard et al., 2020). Intralimb coordination also varies as a function of swim condition, which in return affects temporal parameters of the stroke (Aujouannet et al., 2006). When swim pace increases, the relative time (in percentage) devoted to propulsion (PrP%) typically follows the same trend in proportion to the total duration of the cycle (Chollet et al., 2000; Seifert et al., 2004; Schnitzler et al., 2011a). The trajectory of the hand is also impacted, as lateral–medial trajectory of the hand seems to lose amplitude with speed (de Jesus et al., 2016), the acceleration pattern is modified (Samson et al., 2015b), and the time lag between two propulsive times from the arms [measured with the index of coordination (IdC); Chollet et al., 2000] diminishes significantly. In expert swimmers, these adaptations are employed to maintain swim efficiency constant across swim speed repertoire (Schnitzler et al., 2010; Seifert et al., 2011, 2013; de Jesus et al., 2016). Therefore, it appears that understanding and analyzing expertise in swimming require the comprehension of factors related to propulsive force generation and drag force minimization. In that regard, coordination and propulsion parameters are of particular interest (Costill, 1992).

The rapid development of theoretical research and swim technology (sensors and other portable devices) in recent years helped to get a more in-depth comprehension of swimmers' behavior, as it might potentially capture more data than what is usually done by motion capture systems. Stroking parameters were first examined (Craig et al., 1979, 1985), in parallel with propulsive kinetic parameters (Goldfuss and Arnold, 1971; Yeater et al., 1981). A method to calculate hand force produced in the water using force sensors was validated by Takagi and Wilson (1999) and subsequently improved (Kudo et al., 2013). The advantage of such empirical data over a model-based photometric method is the capacity to directly measure the complex unsteady fluid phenomena occurring during sculling without reconstruction from a putative model (Kudo et al., 2013; Takagi et al., 2014). However, testing took place on an artificial hand, and not in an ecological context (van Houwelingen et al., 2017). Last, all studies analyzing kinetic parameters (e.g., Schleihauf et al., 1983; Takagi and Wilson, 1999; Kudo et al., 2008; Schnitzler et al., 2011a; Seifert et al., 2011; Barbosa et al., 2013; Gourgoulis et al., 2015) focused on the analysis of the time domain (e.g., mean force, peak forces, standard deviation). In contrast, some experimental studies showed that the analysis of force in the frequency domain holds value in explaining the underlying motor control (Slifkin and Newell, 1998, 1999, 2000). Evidence suggests that systems that display more complexity are usually more adaptive to perturbations. This complexity can be assessed through different means; however, measurement of time–series structures of force signal has been widely used (Slifkin and Newell, 1998, 2000; Slifkin et al., 2000; Vaillancourt et al., 2001; Lipsitz, 2002). These authors showed that when these time–series structure changes from periodic/regular to more complex/random, there are related improvements in the quality of system performance. This was evidenced both in a case of a laboratory task (Slifkin and Newell, 1998, 2000; Slifkin et al., 2000) and in the context of system health (e.g., Vaillancourt et al., 2001; Lipsitz, 2002). The increases in time–series complexity are thought to reflect increased system degrees of freedom that allow for greater flexibility in adaptation to system perturbations or to task constraints. One way of assessing time–series complexity is through spectral analysis. A flatter and broader power spectrum (tending toward white noise) reflects increased time–series complexity. In that, examining the breadth of the force spectrum and its evolution at different paces might help to determine whether expert swimmers display more functional adaptability than less capable swimmers.

However, the impact of swim pace and expertise on force development in the frequency domain remains uninvestigated.

To summarize, when modifying swimming pace, adaptations of stroking and kinetic parameters are expected. This can be achieved by increasing stroke rate (SR) and/or SL, or any combination of these parameters (Craig and Pendergast, 1979; Seifert et al., 2004; Huot-Marchand et al., 2005; Potdevin et al., 2006). Finer motor adaptation may also occur, through coordination changes and/or changes in force production, adaptations that may vary according to the level of expertise. We aim to examine swimmer's adaptation to four different swim paces by simultaneously analyzing, stroking, coordination, and kinetic parameters in ecological conditions as a function of three expertise levels. We hypothesized that (i) to swim faster, front crawl swimmers increase both propulsion time and the overall force impulse during a swimming cycle; and (ii) in the frequency domain, expert swimmers are able to maintain the relative contribution of the main harmonics to the overall force spectrum.

## Materials and Methods

### Participants

A convenience sample of 20 male swimmers participated in the present study. We subdivided this group into three distinct categories: low, medium, and high level of expertise, as a function of the percentage of world record in 100 m, they individually reached maximal speed during the test (Table 1). Before the experiment, a brief interview with each swimmer verified the absence of injuries and diseases. We also checked if they were able to swim front crawl. We obtained written informed consent from participants and (where necessary) their parents before testing began. We informed participants of all risks, sources of discomfort, and benefits involved in the study. Procedures were in accordance with the Helsinki Declaration of 1975, and the study was approved in advance by the participating institution's Human Ethics Committee (reference no. 06/190).

**Table 1:** Main characteristics of the participants.

**Expertise level**	**Training/wk (h)**	**Age (y)**	**Weight (kg)**	**Height (cm)**	**BMI (kg/m^**2**^)**	**Hand surface area (cm^**2**^)**	**Maximal speed (m · s^**−1**^)**	**% of world record speed (100 m)**
Low (*n =* 6)	0.5	32.5 ± 4.0	72.5 ± 13.6	174.2 ± 7.0	23.8 ± 3.3	165 ± 25	1.24 ± 0.05	45.4 ± 3.7
Medium (*n =* 6)	4	27.0 ± 7.5	71.5 ± 9.2	178.2 ± 8.4	22.4 ± 1.4	172 ± 16	1.54 ± 0.1	69.3 ± 4.9
High (*n =* 8)	14	18.7 ± 2.9	71.0 ± 4.0	177.6 ± 6.1	22.5 ± 1.8	159 ± 14	1.82 ± 0.05	82.5 ± 2.6

### Data Collection

#### Calculation of v_max_ and Subvelocities

The swim trials took place in a motorized aquatic flume in a temperature- and humidity-controlled laboratory environment. All testing was conducted between 8 and 11 A.M. on weekdays, and participants were instructed to rest the day before and not to change their dietary, hydration, or sleep habits prior to the experiment. All participants were informed they had to complete the trial in front crawl. They performed a standardized 20-min warm-up provided by a coach in the flume before the experiment, which also served as a familiarization period. Prior to the experiment, their maximal swim speed (*v*_max_) in the flume was determined. The water flow was set at a velocity between 0.5, 1.0, and 1.2 m s^−1^ (for low, medium, and high skill level, respectively), and participants were asked to swim as fast as possible over a distance of 5 m. Subsequent swim speed *v*5 was calculated according to Equation (9).

(8) $$\begin{array}{r} v5=5/t \end{array}$$

where *v*5 is the velocity over 5 m relative to the mark on the floor, and *t* is the time to complete 5 m in the flume. To calculate individual maximal swim speed, *v*5 was added to flume's water speed flow according to Equation (8):

(9) $$\begin{array}{r} v_{max}=v_{flow}+v5 \end{array}$$

where *v*_max_ is the maximal swim speed, *v*_flow_ is the water flow speed, and *v*5 is the speed over 5 m relative to the floor. Last, after 20-m rest, the flume was set at the calculated speed, and the participants were instructed to stay above a mark at the bottom of the flume as long as possible. The speed was considered maximal if participant could maintain their position between 10 and 15 s with the head above the mark at the bottom of the flume. The individual results are displayed in Table 2.

**Table 2:** Individual values for maximum swim velocities.

**Subject n^**°**^**	**Expertise**	***V*_**flow**_**	**T_**5m**_**	***V*_**max**_**
1	High	1.2	7.1	1.90
2	High	1.2	8.2	1.81
3	High	1.2	8.2	1.81
4	High	1.2	8.2	1.81
5	High	1.2	8.3	1.80
6	High	1.2	8.3	1.80
7	High	1.2	8.3	1.80
8	High	1.2	10	1.70
9	Medium	1	8.2	1.61
10	Medium	1	8.2	1.61
11	Medium	1	8.3	1.60
12	Medium	1	10	1.50
13	Medium	1	10.4	1.48
14	Medium	1	11.6	1.43
15	Low	0.5	6.4	1.28
16	Low	0.5	6.4	1.28
17	Low	0.5	7.1	1.20
18	Low	0.5	7.1	1.20
19	Low	0.5	7.7	1.15
20	Low	0.5	7.7	1.15

Four individual-specific speeds relative to *v*_max_ (or paces) were determined: pace 1 (70%), pace 2 (80%), pace 3 (90%), and pace 4 (100% of *v*_max_). For paces 1–3, we instructed the swimmers to stay within a delimited zone of 3 m at least 20 s to ensure that they kept following the pace. This duration was reduced to 10 s for pace 4 due to fatigue. To minimize fatigue effects, participants had at least 20-min rest between the determination of *v*_max_ and second part of testing. During this second part, a minimum of 4 min of rest was imposed between paces.

Before each swim bout, the water flow was set at the required speed. The swimmer was then instructed to hold onto a support rope in a streamline position at the center of the flume. The start position was standardized when the swimmer's head was aligned above a mark at the bottom of the flume. The swimmer was considered unable to follow the pace once his head passed a second mark placed 1.5 m behind the first mark. Once the data were collected, the swimmer could then either hold to a rope or go to the side to catch a rail. If any sign of weakness was observed (i.e., difficulty to maintain the pace, swimmer passing the second mark), the experimenter immediately stopped the flume. For security purposes, safety nets were placed 3 m behind the swimmers' feet, which would prevent a collision with the flume vanes behind the swimmer. However, this problem did not occur during our experimentation.

Three underwater 50-Hz digital video cameras were positioned around the flume from two front angles (45° left and right of the swimmer) and a right profile view. The videos and the force signal were synchronized at 50 Hz with the force signal using a digital control unit. More precisely, just before data collection, we pressed a button within the digital control unit that set a trigger that was visible in both signals (i.e., a spike in the force signal, a mark on all videos). Using this signal, we synchronized force and video signal at 50 Hz using Simi motion reality system (Unterschleissheim, Germany) software. From the video, it was therefore easy to distinguish, within the force signal, the portion corresponding to the recovery phase and the portion corresponding to entry, catch, pull, and push phases with an accuracy of 0.02 s.

We used these synchronized videos to quantify SR and SL. We calculated each variable based on three complete representative swim strokes. The SL (in m · cycle^−1^) and SI [(m^2^ · (s · cycle)^−1^] were derived from the mean speed (*v*, in m s^−1^) and the movement frequency value (SR, expressed in Hz). We used Equations 11 and 12 to calculate SL and SI:

(11) $$\begin{array}{rc} \text{SL } & =\;v\;\times \text{ S}{\text{R}}^{-1} \end{array}$$

(12) $$\begin{array}{rc} \text{SI } & =\text{ SL }\times \;v \end{array}$$

#### Coordination Parameters

The mean duration of a complete stroke was the sum of the propulsive and non-propulsive phases. We derived the IdC as the time gap between the propulsion of the two arms as a percentage of the duration of the complete arm stroke cycle.

We divided arm stroke into four distinct phases:

*Phase A: Entry and catch* of the hand in the water, which corresponds to the time between the entry of the hand into the water and the beginning of its backward movement and by a sudden increase in the force developed within the water.

*Phase B: Pull phase*, which corresponds to the time between the end of phase A and its entry into the plane vertical to the shoulder.

*Phase C: Push phase*, which corresponds to the time between the end of phase B and the exit of the hand from the water or a null value obtained on the force graph.

*Phase D: Recovery phase*, which corresponds to the end of phase C and the entry of the hand into the water.

The total duration of these stroke phases was measured by two independent operators with a blind technique for each arm over three complete stroke cycles per pace with a precision of 0.02 s and expressed as a percentage of the duration of a complete arm stroke.

IdC was the mean of IdC_left_ and IdC_right_ (Equations 13 and 14):

(13) $$\begin{array}{l} {\text{IdC}}_{\text{left}}=\\ \left[({\text{Time}}_{\text{end of phase C for left-arm}}\;-{\text{Time}}_{\text{beginning of phase B for right-arm}}\right)\\ \times 100]/{\text{duration}}_{\text{complete cycle}} \end{array}$$

(14) $$\begin{array}{l} {\text{IdC}}_{\text{right}}=\\ \left[({\text{Time}}_{\text{end of phase c right-arm}}{\text{ Time}}_{\text{beginning of phase b for left-arm}}\right)\\ \times 100]/{\text{Duration}}_{\text{complete cycle}} \end{array}$$

The total propulsive phase duration was calculated as the addition of pull-and-push phase duration (in seconds) and also expressed in relative (PrP%) duration, as a percentage of the cycle's time. For each pace, three cycles were analyzed per swim trial, which corresponded with the cycles taken to determine stroke (SR, SL) and coordination (IdC, propulsive phase) parameters.

#### Kinetic Parameters

**Figure 1:**
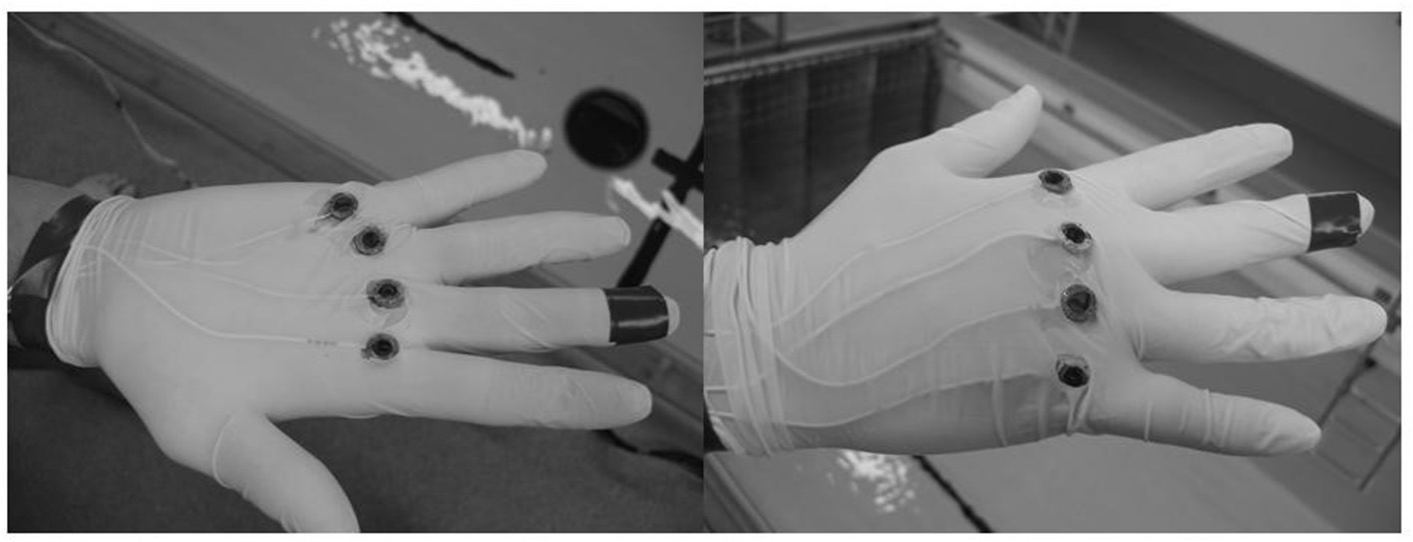
Locations of the force sensors over the hand.

The methodology used to determine kinetic parameters follows the methods from Takagi and Wilson (1999). On the swimmers' preferential hand, we glued four pairs of monoaxial pressure sensors (Kyowa, Tokyo, Japan, see Figure 1) to the surface of a glove on both the palmer and dorsal sides of metacarpophalangeal II, III, IV, and V. The load cell can transduce oscillations of frequencies over a range from 0 to 1,000 Hz. Force applied to the load cell resulted in changes in the electrical resistance of strain gauges housed in the load cell. The sensors were connected via a series of wires to a 12-entry amplifier, connected itself to a computer to record the force data, and calibrated in the water. We measured forces in units of 0.001 N (0.1 g). The sensors were paired by metacarpus; for example, the sensor of the palmer side of metacarpus II (PMII) was paired with metacarpus II of the dorsal side (DMII), as shown in Figure 1.

The hand plane area was measured. Each swimmer had their palmar face of the hand scanned, thumb adducted, and fingers fully extended and packed together. Then, we computed this area using Mesurim 3.3 software.

We measured pressure differential (*P*_A_) so that in the absence of movement: *P*_A_ = PMII – DMII = 0. We calculated this difference in pressure for metacarpus III (P_B_), metacarpus IV (P_C_), and metacarpus V (P_D_). After this first calibration of the sensor pairs in water, we were able to obtain the mean moving pressure using the Equation 15 (Takagi and Wilson, 1999).

(15) $$\begin{array}{l} P_{mean}=0.045P_{A}\;+0.186\;P_{B}\;+0.554\;P_{C}\;+0.013\;P_{D}\\ \;+7.558 \end{array}$$

The obtained value was then multiplied by the hand plane area previously determined (m^2^) to calculate the resultant propulsive peak-medium force.

Because of technical limitations, we could only measure the force developed by one hand using Equation 15. In order to standardize conditions, the dominant hand of each subject was chosen for the collection of kinetic data. We analyzed force output over six consecutive swim cycles in both time and frequency domains, which are two complementary methods to examine kinetic parameters (Prandoni and Vetterli, 2008). In the time domain, and with the help of the swim phases determined with the video analysis, we computed the force impulse during propulsive phases per cycle, which captures the magnitude of the fluctuations. In the frequency domain, we made a spectral analysis using fast Fourier transforms (FFTs), which measure the structure of the variability (Slifkin and Newell, 1999).

**Figure 2:**
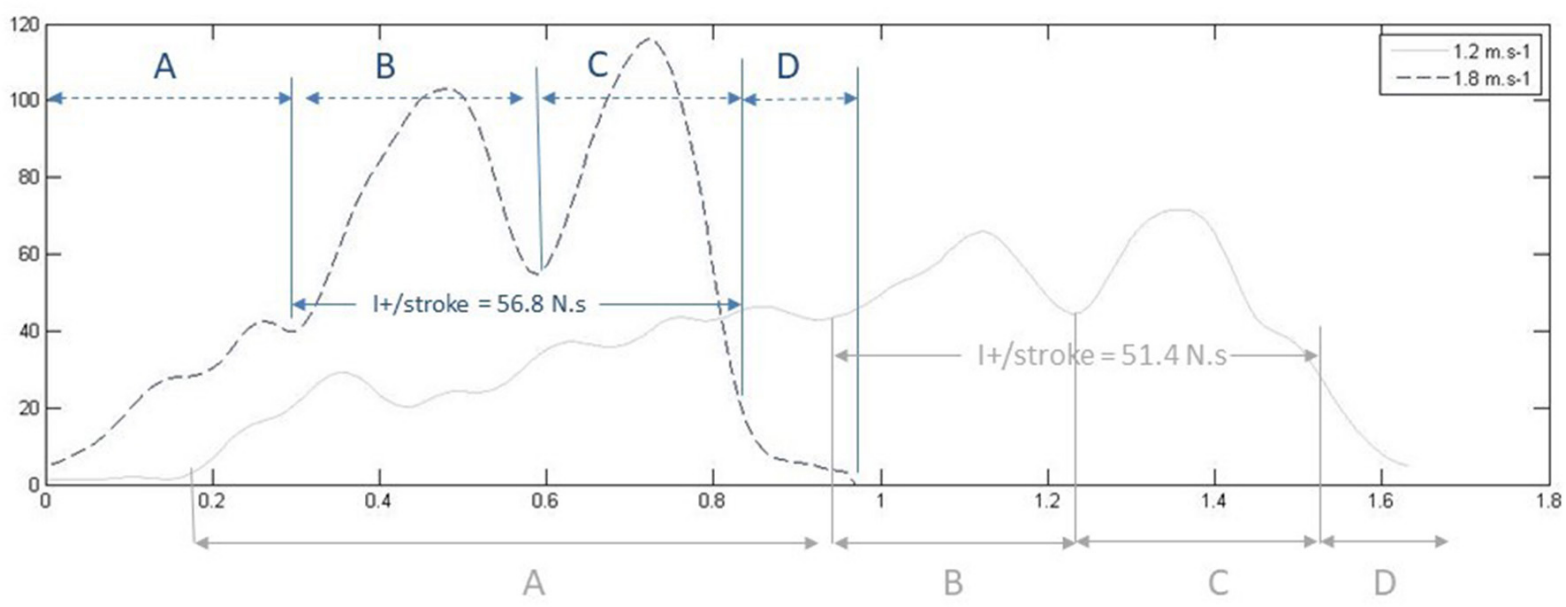
Example force time series from a national level swimmer at two different paces (V1 = 1.2 m.s^−1^, 70% vs. V4 = 1.8 m.s^−1^, 100%), and the corresponding duration of the swim phases for each pace. The graph illustrates how two cycles can have comparable overall impulses (I^+^/stroke) whereas Fpull and Fpush are of higher magnitude at V4 compared to V1.

We superimposed both force and video signals on a single graphical user interface to calculate force impulse, at the frequency of 50 Hz. We reconstructed the force signal to only take into consideration the force developed during propulsive time (pull-and-push phases) to calculate the propulsive impulse (*I*^+^). We used the force graph to measure peak pull and peak push force. Figure 2 shows an example of two force curves and the correspondence with the swim phases (determined by video, not shown in this figure for the sake of clarity).

To minimize the error in calculating kinetic parameters, we examined a period of 6 cycles as a whole (Payton and Bartlett, 1995). We used a MATLAB signal processing toolbox (MATLAB 16, MathWorks, Natick, MA) routine to perform an FFT. The power spectrum of each trial was divided into 50 equal bins, ranging from 0 to 10 Hz. On all curves, we manually identified three main peaks. Each of them represents a specific source of variation within the force signal. The power in each of these three specific frequencies bin (Y1, Y2, and Y3) represented the portion of total power in the overall amplitude of force output oscillation that could be attributed to the frequency specified by each bin. Y1 was the fundamental frequency and typically occurred in a 0–3.33-Hz range. Y2 was the second in magnitude and occurred in 3.34–6.66-Hz range. Y3 was the smallest in magnitude and occurred in the 6.67–10-Hz range. To provide a measure of the spread of power in the power spectrum, we divided the peak power by the total power in the power spectrum. Therefore, we calculated the ratio between each specific frequency and the total power (obtained by numerical integration of the power spectrum curve) to examine the modification of force output according to pace [see Slifkin and Newell (1999)].

We used means and standard deviations to summarize the dependent variables as a function of expertise level. The assumed Gaussian distribution of the data was verified by the Shapiro-Wilk test and the homogeneity of variance using Bartlett test. Mixed-design analysis of variance (ANOVA) subject [repeated measure] × 4 pace levels [70, 80, 90, and 100% of *v*_max_ ] × 3 expertise levels [low, medium, high] compared the mean values for each variable. Tukey *post-hoc* tests were run to detect significant differences between pairs of condition means. Partial *η*^2^ and its 95% confidence interval were used to estimate effect sizes. We set the threshold for significance at the 0.05 level of confidence. We used R software (R core team, 2017) for the statistical analysis.

## Results

The results of the three-way ANOVAs for different variables are arranged in Table 3 (stroking parameters), Table 4 (coordination parameters), and Table 5 (kinetic parameters). To swim faster, all participants increased the SR and the SI (Table 3). At pace 2 (e.g., 80% of v_max_), only high-level swimmers were able to increase SR and SL simultaneously. High-level swimmers were able to maintain both high SR and SL, whereas a medium-level swimmer had a higher SL.

**Table 3:** Stroking parameters according to pace and expertise level.

**Level**	**Pace**	**Speed (m · s^**−1**^)**	**Stroke index [m^**2**^ · (s · cycle)^**−1**^]**	**Stroke rate (cycle · min^**−1**^)**	**Stroke length (m · cycle^**−1**^)**
High	1	1.29 ± 0.01^b,c,d^	2.81 ± 0.16^d^	35.75 ± 2.24^c,d^	2.18 ± 0.13
High	2	1.42 ± 0.04^a,c,d^	3.14 ± 0.10	38.30 ± 2.91^c,d^	2.22 ± 0.11
High	3	1.59 ± 0.09^a,b,d^	3.40 ± 0.45	44.70 ± 2.35^a,b^	2.14 ± 0.18
High	4	1.80 ± 0.05^a,b,c^	3.60 ± 0.26^a^	54.09 ± 3.99^a,b^	2.00 ± 0.13
Medium	1	1.02 ± 0.18^b,c,d^	2.16 ± 0.47^d^	29.93 ± 7.62^d^	2.11 ± 0.34
Medium	2	1.2 ± 0.11^a,c,d^	2.60 ± 0.48	34.15 ± 7.30	2.17 ± 0.37
Medium	3	1.33 ± 0.07^a,b,d^	2.86 ± 0.54	38.55 ± 8.46	2.15 ± 0.43
Medium	4	1.53 ± 0.07^b,c,d^	3.11 ± 0.47^a^	46.05 ± 6.16^a^	2.03 ± 0.28
Low	1	0.75 ± 0.08^b,c,d^	1.13 ± 0.29^d^	30.63 ± 3.58^d^	1.49 ± 0.26
Low	2	0.91 ± 0.08^a,c,d^	1.41 ± 0.31	36.23 ± 5.44^d^	1.54 ± 0.27
Low	3	1.01 ± 0.07^a,b,d^	1.62 ± 0.32	38.64 ± 4.74	1.59 ± 0.24
Low	4	1.21 ± 0.06^b,c,d^	1.94 ± 0.29^a^	45.90 ± 6.05^a,b^	1.61 ± 0.22
Pace effect		^*^ *F*_(3, 68)_ = 27.39 *p* < 0.001, *ηp*^2^ = 0.52 CI [0.36–0.60]	^*^ *F*_(3, 68)_ = 7.09 *p* < 0.01, *ηp*^2^ = 0.20 CI [0.07–0.32]	^*^ *F*_(3, 68)_ = 34.36 *p* < 0.001, *ηp*^2^ = 0.06 CI [0.43–0.65]	NS *F*_(3, 68)_ = 0.64 *p* = 0.6, *ηp*^2^ = 0.01 CI [0.00–0.07]
Expertise effect		^⊙^ 1, 2, 3 *F*_(2, 68)_ = 37.04 *p* < 0.001, *ηp*^2^ = 0.50 CI [0.34–0.58]	^⊙^ 1, 2, 3 *F*_(2, 68)_ = 33.98 *p* < 0.001, *ηp*^2^ = 0.48 CI [0.31–0.56]	^⊙^ 1, 2 *F*_(2, 68)_ = 7.3 *p* < 0.001, *ηp*^2^ = 0.15 CI [0.04–0.26]	^⊙^ 2, 3 *F*_(2, 68)_ = 19.94 *p* < 0.001, *ηp*^2^ = 0.34 CI [0.18–0.45]
Pace × expertise		NS *F*_(6, 68)_ = 0.09 *p* = 0.99, *ηp*^2^ = 0.00 CI [0.00–0.09]	NS *F*_(6, 68)_ = 0.12 *p* = 0.99, *ηp*^2^ = 0.07 CI [0.00–0.001]	NS *F*_(6, 68)_ = 0.35 *p* = 0.90, *ηp*^2^ = 0.05 CI [0.00–0.04]	NS *F*_(6, 68)_ = 0.36 *p* = 0.90, *ηp*^2^ = 0.05 CI [0.00–0.04]

⊙ *significant difference between 1 (high and medium), 2 (high and low), and 3 (medium and low)*.

* *significant difference among paces*.

*Within expertise level, significant difference with a: pace 1, b: pace 2, c: pace 3, d: pace 4*.

*NS, non**-**significant difference*.

**Table 4:** Coordination parameters according to pace and expertise level.

**Expertise level**	**Pace**	**Propulsive phase duration (s)**	**IdC (%)**	**Propulsive phase duration (%)**	**A (%)**	**B (%)**	**C (%)**	**D (%)**
High	1	0.83 ± 0.11	−3.4 ± 3.2^d^	48.7 ± 5.0^d^	29.1 ± 6.8^d^	25.3 ± 3.3^d^	23.4 ± 5.0	22.2 ± 2.3
High	2	0.76 ± 0.07	−2.3 ± 4.1	47.6 ± 3.6	27.8 ± 7.0	24.9 ± 3.0	22.7 ±± 3.9	24.6 ± 4.2
High	3	0.63 ± 0.05^a^	−1.6 ± 3.2	47.3 ± 2.3	28.6 ± 3.6	25.9 ± 1.9	21.8 ± 1.6	23.7 ± 2.6
High	4	0.61 ± 0.12^a^	6.4 ± 5.6^a^	56.7 ± 6.9^a^	21.1 ± 7.1^a^	30.3 ± 5.5^a^	26.4 ± 3.8	22.2 ± 3.1
Medium	1	0.87 ± 0.19	−8.1 ± 3.8^d^	41.8 ±± 3.5^d^	38.8 ± 4.1^d^	18.5 ± 3.8^d^	22.3 ± 3.8	20.4 ± 3.1
Medium	2	0.77 ± 0.12	−6.9 ± 5.4	42.8 ± 3.8	36.4 ± 4.8	20.4 ± 4.1	22.3 ± 2.4	20.8 ± 2.2
Medium	3	0.70 ± 0.12^a^	−5.2 ± 3.5	44.3 ± 3.8	33.9 ± 6.0	21.6 ± 2.9	22.6 ± 2.6	21.8 ± 2.9
Medium	4	0.60 ± 0.06^a^	−2.5 ± 4.5^a^	48.4 ± 6.5^a^	28.3 ± 9.6^a^	24.4 ± 4.9^a^	24.0 ± 2.6	23.3 ± 3.8
Low	1	0.98 ± 0.17	1.2 ± 6.4^d^	49.9 ± 6.4^d^	27.3 ± 8.5^d^	23.4 ± 2.8^d^	26.4 ± 4.3	22.8 ± 3.9
Low	2	0.88 ± 0.17	3.2 ± 7.5	51.6 ± 6.8	25.3 ± 8.7	25.1 ± 4.3	26.5 ± 3.6	23.1 ± 3.1
Low	3	0.80 ± 0.13	4.2 ± 9.2	52.8 ± 10.0	22.9 ± 10.7	26.6 ± 4.9	26.2 ± 6.1	24.3 ± 2.1
Low	4	0.76 ± 0.13	7.5 ± 7.0^a^	57.3 ± 7.6^a^	18.8 ± 6.2^a^	30.0 ± 5.0^a^	27.4 ± 3.5	23.8 ± 2.6
Pace effect		^*^ *F*_(3, 68)_= 6.95 *p* < 0.001, *ηp*^2^ = 0.23 CI [0.07–0.31]	^*^ *F*_(3, 68)_= 6.60 *p* < 0.001, *ηp*^2^ = 0.22 CI [0.06–0.31]	^*^ *F*_(3, 68)_= 6.95 *p* < 0.001, *ηp*^2^ = 0.23 CI [0.7–0.31]	^*^ *F*_(3, 68)_= 5.69 *p* < 0.001, *ηp*^2^ = 0.20 CI [0.04–0.28]	^*^ *F*_(3, 68)_= 7.75 *p* < 0.001, *ηp*^2^ = 0.25 CI [0.08–0.33]	NS *F*_(3, 68)_= 1.53 *p* > 0.2, *ηp*^2^ = 0.06 CI [0.0–0.12]	NS *F*_(3, 68)_= 0.79 *p* > 0.5 *ηp*^2^ = 0.03 CI [0.0–0.09]
Expertise effect		^⊙^ 2, 3 *F*_(2, 68)_= 37.04 *p* < 0.001, *ηp*^2^ = 0.52 CI [0.34 0.58]	^⊙^ 1, 2, 3 *F*_(2, 68)_= 19.03 *p* < 0.001, *ηp*^2^ = 0.35 CI [0.18–0.44]	^⊙^ 1, 3 *F*_(2, 68)_= 37.04 *p* < 0.001, *ηp*^2^ = 0.52 CI [0.34–0.58]	^⊙^ 1, 3 *F*_(2, 68)_= 13.88 *p* < 0.001, *ηp*^2^ = 0.28 CI [0.12–0.37]	^⊙^ 1, 3 *F*_(2, 68)_= 13.49 *p* < 0.001, *ηp*^2^ = 0.28 CI [0.11–0.37]	^⊙^ 2, 3 *F*_(2, 68)_= 6.63 *p* < 0.01, *ηp*^2^ = 0.16 CI [0.03–0.25]	NS *F*_(2, 68)_= 2.5 *p* < 0.09, *ηp*^2^ = 0.06 CI [0.0–0.14]
Pace × Expertise		NS *F*_(6, 68)_= 0.37 *p* = 0.99, *ηp*^2^ = 0.03 CI [0.00 0.04]	NS *F*_(6, 68)_= 0.42 *p* = 0.86, *ηp*^2^ = 0.03 CI [0.00–0.04]	NS *F*_(6, 68)_= 0.37 *p* = 0.99, *ηp*^2^ = 0.03 CI [0.00–0.04]	NS *F*_(6, 68)_= 0.15 *p* = 0.98, *ηp*^2^ = 0.01 CI [0.00–0.01]	NS *F*_(6, 68)_= 0.20 *p* = 0.98, *ηp*^2^ = 0.01 CI [0.00–0.01]	NS *F*_(6, 68)_= 0.34 *p* < 0.91 *ηp*^2^ = 0.02 CI [0.00–0.04]	NS *F*_(6, 68)_= 0.78 *p* < 0.59, *ηp*^2^ = 0.00 CI [0.00–0.09]

* *Significant difference among paces*.

⊙ *Significant difference between 1 (high and medium level), 2 (high and low level), and 3 (medium and low level)*.

*Within expertise level, significant difference with a: pace 1, b: pace 2, c: pace 3, d: pace 4*.

**Table 5:** Kinetic parameters according to pace and expertise level.

**Level**	**Pace**	**Force impulse/cycle (N · s)**	**Pull Force (N)**	**Push force (N)**	**Y1/tot power**	**Y2/tot power**	**Y3/tot power**
High	1	63.4 ± 14.4	58.8 ± 12.1	73.7 ± 17.8	0.28 ± 0.11	0.11 ± 0.05^d^	0.016 ± 0.007
High	2	68.3 ± 20.2	59.0 ± 12.1	77.6 ± 16.0	0.30 ± 0.15	0.07 ± 0.04	0.013 ± 0.004
High	3	65.1 ± 21.2	66.5 ± 13.2	80.1 ± 13.2	0.32 ± 0.12	0.06 ± 0.04	0.013 ± 0.005
High	4	75.4 ± 22.9	74.5 ± 18.4	84.9 ± 22.9	0.25 ± 0.09	0.03 ± 0.02^a^	0.013 ± 0.005
Medium	1	63.2 ± 23.3	55.9 ± 26.5	65.9 ± 26.5	0.19 ± 0.10	0.09 ± 0.05	0.022 ± 0.016
Medium	2	70.1 ± 17.6	68.8 ± 27.2	79.0 ± 23.1	0.25 ± 0.13	0.11 ± 0.05	0.018 ± 0.012
Medium	3	80.6 ± 20.2	73.424.8	90.8 ± 15.5	0.28 ± 0.14	0.07 ± 0.03	0.018 ± 0.008
Medium	4	82.6 ± 15.1	82.2 ± 30.1	97.9 ± 17.3	0.36 ± 0.25	0.08 ± 0.06	0.018 ± 0.013
Low	1	78.2 ± 6.8	51.6 ± 16.5	69.6 ± 20.3	0.24 ± 0.13	0.04 ± 0.05	0.005 ± 0.005
Low	2	87.9 ± 5.4	57.4 ± 14.0	80.2 ± 23.1	0.28 ± 0.11	0.04 ± 0.03	0.002 ± 0.015
Low	3	86.1 ± 10.5	60.1 ± 9.4	85.0 ± 23.4	0.26 ± 0.32	0.02 ± 0.01	0.006 ± 0.005
Low	4	83.7 ± 20.9	63.4 ± 17.1	82.6 ± 27.3	0.27 ± 0.27	0.02 ± 0.01	0.007 ± 0.008
Pace effect		NS *F*_(3, 68)_= 1.64 *p* < 0.18, *ηp*^2^ = 0.06 CI [0.0–0.31]	^*^ *F*_(3, 68)_= 3.06 *p* < 0.03, *ηp*^2^ = 0.11 CI [0.07–0.31]	^*^ *F*_(3, 68)_= 3.09 *p* < 0.03, *ηp*^2^ = 0.11 CI [0.07–0.31]	NS *F*_(3, 68)_= 0.49 *p* < 0.61, *ηp*^2^ = 0.02 CI [0.0–0.02]	^*^ *F*_(3, 68)_= 3.45 *p* < 0.02, *ηp*^2^ = 0.13 CI [0.01–0.21]	NS *F*_(3, 68)_= 1.04 *p* < 0.37, *ηp*^2^ = 0.04 CI [0.0–0.09]
Expertise effect		^⊙^ 2, 3 *F*_(2, 68)_= 5.23 *p* < 0.008, *ηp*^2^ = 0.13 CI [0.02–0.22]	NS *F*_(2, 68)_= 2.25 *p* < 0.11, *ηp*^2^ = 0.06 CI [0–0.13]	NS *F*_(2, 68)_= 0.34 *p* < 0.7 *ηp*^2^ = 0.01 CI [0.0–0.04]	NS *F*_(2, 68)_= 0.2 *p* < 0.80, *ηp*^2^ = 0.005 CI [0.34–0.58]	^⊙^ 2, 3 *F*_(2, 68)_= 8.43 *p* < 0.001, *ηp*^2^ = 0.20 CI [0.34–0.58]	^⊙^ 2, 3 *F*_(2, 68)_= 5.09 *p* < 0.009, *ηp*^2^ = 0.13 CI [0.34–0.58]
Pace × expertise		NS *F*_(6, 68)_= 0.47 *p* < 0.99*,ηp*^2^ = 0.03 CI [0.00–0.04]	NS *F*_(6, 68)_= 0.24 *p* < 0.96, *ηp*^2^ = 0.02 CI [0.00–0.01]	NS *F*_(6, 68)_= 0.45 *p* < 0.84, *ηp*^2^ = 0.03 CI [0.00–0.04]	NS *F*_(6, 68)_= 0.52 *p* < 0.79, *ηp*^2^ = 0.03 CI [0.00–0.04]	NS *F*_(6, 68)_= 0.55 *p* < 0.7, *ηp*^2^ = 0.03 CI [0.00–0.05]	NS *F*_(6, 68)_= 0.63 *p* < 0.7, *ηp*^2^ = 0.05 CI [0.00–0.06]

* *Significant difference with a: pace 1, b: pace 2, c: pace 3, d: pace 4*.

⊙ *Significant difference between 1 (high and medium), 2 (high and low), and 3 (medium and low)*.

When increasing swim pace, participants decreased the catch phase and increased pull phase duration, which increases propulsive phase duration in percentage and, subsequently, the IdC. Low-level swimmers had significantly longer propulsive phase duration (both in absolute and relative duration) and higher IdC, as compared to high- and medium-level swimmers. Across paces, catch phase (A) decreased significantly, whereas pull phase (B) increased significantly. Medium-level swimmers displayed significantly higher values for catch phase (A) and lower values for pull phase (B) as compared to both high and low levels. Finally, high-level swimmers presented significantly higher values for pull phase (C) as compared to both medium and low levels.

To swim at faster speeds, participants tend to increase pull and push peak force, whereas the second harmonic of the force signal decreases significantly. High- and medium-level swimmers both exhibit higher values in these second (Y2) and third (Y3) harmonics, and also lower force impulse throughout the trial as compared to low level of swimmers.

## Discussion

Based on Newell (1986) constraint-led approach, the objective of this study was to provide a systemic view of how swimmers adapt to water flow (environmental constraints) in front crawl (task constraint) as a function of expertise (an organismic constraint). The results show that to swim faster, participants increase SR, IdC, propulsive phase duration, and force peak and modify the second harmonic of the force signal in the power spectrum. Higher SI and SL characterize high-level swimmers, whereas high-frequency contributions of the force signal were not shown by the low-level swimmers.

To swim at different swim paces, swimmers modify stroking parameters. The SI, in particular, increases significantly in all expertise levels across pace. As swim speed is the product of SR and SL, this modification is mainly explained by an increase in SR, whereas SL does not change significantly. Hence, swim speed is mainly controlled by modifying the SR, in accordance with past studies (Craig and Pendergast, 1979; de Jesus et al., 2016). However, coordination parameters show other adjustments occur as pace increases, as IdC and PrP% significantly increase over pace. These results are consistent with the current literature dealing with stroking and coordination parameters: when swim pace goes from low to high speed, there is a significant increase in PrP% and IdC toward a “superposition” mode, as catch phase (A) decreases, while pull phase (B) increases (Chollet et al., 2000; Seifert et al., 2004, 2011; Schnitzler et al., 2011a). According to Samson et al. (2015b), this modification in catch phase relative duration is bound to ensure the optimal horizontal balance of the body: at a low swimming speed, the hand stretches horizontally, and the resulting streamlining not only produces minimum energy expenditure and drag, but also optimizes the propulsive action of the opposite arm, whereas at high speed, the drag force generated during catch phase is higher but shorter, allowing high propulsive forces to be developed during the subsequent phases. In line with previous findings (Chollet et al., 2000; Seifert et al., 2004, 2011; Schnitzler et al., 2011a), these results show that swimmers of all levels were mostly flexible as they increased their IdC to increase their speed. Seifert et al. (2011) demonstrated that stroke cycle changes in arm coordination are linked to variations in aquatic resistance, as more overlapping of the two propulsion phases enables the swimmer to achieve higher swimming speeds.

Interestingly, this study also shows how these speed adaptations differ among expertise levels. In what concerns stroking parameters, SL and SI magnitudes are closely associated with expertise level, revealing underlying differences in swim efficiency (Costill et al., 1985; Craig et al., 1985; Toussaint, 1990; Seifert et al., 2011). These differences across expertise levels were mainly due to longer A and shorter B phase relative duration in medium-level swimmers. Consequently, the IdC values had a U-shaped relationship, with low- and high-level swimmers displaying higher values than average. Indeed, Dadashi et al. (2016) showed that IdC magnitude only predicts swimming performance in homogeneous expertise groups. The present data show that low-level swimmers start their propulsion early by shortening the catch phase, which might result in a less efficient positioning of the hand during the propulsive phase. As shown by Koga et al. (2020), inefficient propulsion is associated with a low angle of attack at the end of the catch phase. This is confirmed by the fact that at low speeds, the impulse force is higher, and the pull-and-push forces are similar to those of medium- and high-level swimmers. According to this reasoning, medium-expertise-level swimmers take more time than low-expertise-level swimmers to position their hand to improve the efficiency of the propulsion phase, whereas high-expertise-level swimmers seem to be able to combine a short catch phase duration with high propulsion phase efficiency. However, these proposals have yet to be confirmed experimentally, as the present study did not measure the efficiency of the propulsion phase. In line with previous findings (Schnitzler et al., 2010), low-level swimmers in our study exhibit higher IdCs. Seifert et al. (2014) suggested that low-expertise swimmers used an inefficient superposition mode, as they “slip” through the water, that is, producing insufficient force while increasing swim frequency. It appears that low-level swimmers “waste” much of their force production imparting kinetic energy of surrounding water, with force impulses significantly higher than high- and medium-expertise groups. Ultimately, these findings support Seifert et al.'s (2011) assertion that “a relative lack of skill and technique could lead to lower efficiency of propulsion generation.”

With regard to kinetic data, prior research had identified different adaptive modes to changes in swimming speed. Using hand paddles, Gourgoulis et al. (2008) showed that increasing propelling surfaces resulted in a concomitant increase in both force and maximal speed. According to Tsunokawa et al. (2019), this was attributable to an increase on Froude efficiency when using paddles. However, Samson et al. (2015a) showed that propulsive hand forces did not vary significantly across swim paces. Furthermore, Koga et al. (2020) showed that the adoption of overmaximal SR did not help swimmers to reach higher swim speed, as this led to lower angles of attack, which induced lower hand propulsive force. Therefore, the increase in swimming pace is explained by the swimmer's capacity to maintain propulsive phases on higher stroke frequency rather than increasing force generation by orienting the hand in a favorable manner before the propulsive phases begin. Our results are in line with these studies, as force impulse during propulsive phases did not change significantly across paces, but low-expertise swimmer exhibited shorter catch phase as compared to medium-level swimmers. It is worth noting that pull and push peak forces increase, which indicates that adaptation nonetheless occurs at kinetic level. We analyzed force impulse as the numerical integration of the propulsive time duration of each cycle. As stroke frequency increases, the total duration of this time decreases, so without an adaptation, force impulse should follow the same trend. In line with Samson et al. (2015a), the fact that push, pull, and peak forces increase with speed suggests that to maintain these force impulses across different speeds, participants have to increase the absolute force they apply to water and reach this peak more quickly, thus delivering more power to the water during propulsive phases, which explains why the impulse per cycle did not decrease. This might explain why Morouço et al. (2018) found that intracyclic force variation increased with swim speed in tethered swimming conditions. Interestingly, low- and medium-level swimmers had similar SR. If the athletes who produce a greater speed should increase the absolute force they apply to water, the impulse of the medium-expertise level should be greater. This is not the case because the impulses of low-level swimmers are greater than those of medium- and high-level swimmers, suggesting that it is not generally increased force production but rather swimming efficiency that is the key to differentiating between levels of expertise.

We aimed to extend these kinetic analyses and examine measures of the structure of force variability through the analysis of the power spectrum of the force–time series. Spectral analysis decomposes a signal into its component frequencies so that the power assigned to each frequency in the spectral profile provides an index of the portion of total amplitude variability that can be attributed to each frequency. A modification in the profile spectrum provides insight about the frequency structure. Here, the power spectrum exhibited three clear peaks within the 0–12-Hz bandwidth. In each case, the first peak corresponded to the stroke frequency. What represents the second and third peak needs to be determined experimentally. Our results show that increasing swim pace modifies the relative duration of each of these phases. In the same vein, Samson et al. (2015a) outlined that the acceleration pattern of the hand changed with swim speed. Hence, the second peak could represent the modification of the propulsive vs. non-propulsive phase ratio. In what concerns the third peak, several authors pointed out that there was also a variation within the propulsive phase (Schleihauf et al., 1983; Monteil et al., 1994), which could be explained by the change in orientation between the pull and the push phase. This variation occurs at a higher frequency within the force signal, and its importance in explaining the overall signal could be represented by the third harmonic. Our data show that the power associated with the second harmonic decreases across pace in all expertise levels, which is consistent with the coordination data showing that propulsive phase represents ~50% of the total at pace 1 to more than 67% at pace 4 in both high- and low-expertise levels. Our data show that the increase in average force is due to more frequent impulses, whereas coordination flexibility helps to maintain individual impulses constant, whatever the expertise level. It is interesting to note that in the Neptune and Herzog (2000) study, this flexibility occurs between muscles rather than within muscles, as these authors showed on a cycling task that pacing-related adaptations occurred through the magnitude of the electromyographic response rather than through a change in intramuscular coordination. These data were not available in the present study, but whether behavioral adaptability responses are specific to exercise mode is a worthy question for future research to address directly.

The examination of the kinetic frequency domain introduces new insight into swim expertise. According to our data, high- and medium-expertise swimmers exhibit higher second and third harmonic components, but only high-expertise swimmers are capable of modifying their second harmonic significantly with pace. This suggests a flatter and broader power spectrum as potential indicators of increased complexity within the force time–series signal. That might reflect the availability of more degrees of freedom in an expert system. Interestingly, it appears that this characteristic within the force spectrum, especially at high frequency, might be a relevant feature to characterize expertise.

Taken together, these novel results suggest that, independently of expertise, the modification of inter- and intra-arm coordination helps to maintain force impulses despite the shorter absolute duration of swim cycles. However, some limitations exist in this study. First, we only measured average force produced *F*_av_, not propulsive force *F*_d_, as the sensors were not oriented in space to detect the application of propulsive force. Second, we were not able to account for a complete description of the force development, as forces were measured at only one hand, whereas force generation patterns involves all the arms (Toussaint and Truijens, 2005). Third, active drag could not be measured, so whether the difference between skill levels was due to higher propulsive force, lower drag, or any combination of the two remains inconclusive. Fourth, a glove on only one hand could have an impact on performance. The glove could affect propulsion asymmetrically and affect coordination, as well as change the perception of water. However, we were still able to outline significant adaptations both at stroking and kinetic parameters, meaning they could be even larger in other settings. Fifth, because of technical limitations, only the force signal corresponding to one hand could be accurately measured, and we could not account for the role of the legs. This is problematic in a sense that asymmetries in arm force production are frequent, although better swimmers tend to be less asymmetric (Dos Santos et al., 2013). Sixth, the spectral analysis used in the current study differs from the usual analyses aimed at assessing time–series complexity. In the current study, three points were considered (Y1, Y2, and Y3). In contrast, in studies aimed at assessing time–series complexity, an assessment of the whole power spectrum is made. Last, these measurements took place in a flume, which modifies the kinematics of the stroke. As Guignard et al. (2019, 2020) recently pointed out, the action of the arms is impacted by the fluid flow in a flume, which constrains the action possibilities more than in a swimming pool. Future studies should provide means to estimate simultaneously the forces produced by both hands to provide a more accurate measurement of swim efficiency, as well as intralimb and interlimb coordination parameters. Additionally, it would be of interest to contrast whether behavioral adaptability to common features such as speed change is specific to exercise modes (such as swimming) or if they have general transferable properties as a function of the environment, whether it is terrestrial or aquatic.

Despite these limitations, this study was an important first step toward providing a simultaneous analysis of stroking, coordination, and kinetic parameters in an ecological context of swimming. It was also the first to examine force dynamics both in temporal and frequency domains. For the first time in front crawl swimming, we were able to examine the spectral content of the force development, which gives an insight into intrasegmental coordination, as outlined by Slifkin and Newell (1999). We identified three main frequencies in the spectrograms, in line with early studies about force development in front crawl (Yeater et al., 1981), but we showed that medium and high expertise levels exhibited a flatter and more broadband spectral content, but also that the adaptation across pace occurs only in high-expertise swimmers for the third harmonic.

## Conclusion

This study proposed new insights into how swimmers of different skill levels adapt to front crawl swimming at different paces. There are implications in not only sports scientists, but also practitioners and coaches. The main results showed three different levels to take into consideration to perform such investigations: stroking, which expresses the result of the underlying motor control strategy used; coordination, which accounts for this motor control strategy; and kinetic levels, which shows how this motor control leads to force production. Continuing to explore the relationship between those three levels would be of interest in future work. Also, we surmised that these investigations should be carried out not only in the temporal but also in the frequency domain. Finally, to swim at different paces, participants across skill levels shared common characteristics: they all exhibited flexibility, notably in the stroking and the coordination levels. But only the more skilled swimmers were capable of finer intralimb coordination adjustments. In that, stroking, coordination, and kinetic parameters offer promising perspectives in characterizing not only expertise but also the evolution of motor adaptation at an individual level.

## Data Availability Statement

The raw data supporting the conclusions of this article will be made available by the authors, without undue reservation.

## Ethics Statement

The studies involving human participants were reviewed and approved by otago university ethic committee reference number: 06/190. The patients/participants provided their written informed consent to participate in this study.

## Author Contributions

All authors listed have made a substantial, direct and intellectual contribution to the work, and approved it for publication.

## Conflict of Interest

The authors declare that the research was conducted in the absence of any commercial or financial relationships that could be construed as a potential conflict of interest.
